# Weight loss, glycolipid profile changes in type 2 diabetes patients after esophagectomy: a propensity score matching analysis

**DOI:** 10.1007/s00464-024-10852-5

**Published:** 2024-05-09

**Authors:** Jingrong Yang, Jiabin Lai, Xiangrui Chen, Wenxuan Xia, Yaxin Li, Jialei Huang, Yu Wang

**Affiliations:** 1 Department of Cardiothoracic Surgery, The 900th Hospital of Joint Logistic Support Force, PLA, Fuzong Clinical Medical College of Fujian Medical University, Fuzhou, 350025 Fujian People’s Republic of China; 2 The School of Basic Medical Sciences, Fujian Medical University, Fuzhou, 350122 Fujian People’s Republic of China; 3 Department of General Surgery, Fuzong Clinical Medical College of Fujian Medical University & Dongfang Hospital of Xiamen University & The 900th Hospital of Joint Logistics Support Force, Fuzong Clinical Medical College of Fujian Medical University, No.156 North West Second Ring Road, Fuzhou, 350025 Fujian People’s Republic of China

**Keywords:** Esophagectomy, Type 2 diabetes mellitus, Weight loss, Glycolipid profile

## Abstract

**Background:**

Type 2 diabetes mellitus (T2DM) is a common co-morbidity in patients who receive esophagectomy and has unfavorable effects on glucose and lipid metabolism in patients. This study examines how weight and glycolipid metabolism change in patients with T2DM following esophagectomy.

**Methods:**

This retrospective, one-center, observational analysis with a propensity score matching analysis (PSM) included 114 patients who underwent esophageal surgery in the Department of Cardiothoracic Surgery, the 900th Hospital of Joint Logistic Support Force from 2017 to 2020, which were separated into T2DM group and Non-T2DM group. Weight, body mass index (BMI), fasting plasma glucose (FPG), triglycerides (TG), total cholesterol (TC), high-density lipoprotein (HDL), and low-density lipoprotein (LDL) were measured and analyzed before and after the operation.

**Results:**

Two groups showed similar reductions in weight and BMI after surgery. In the T2DM group, weight decreased from 63.10(10.31) before surgery to 55.10(11.60) kg at 6 months (P < 0.001) with BMI decreasing from 22.67 (2.90) to 19.77 (3.48); While in the Non-T2DM group, weight decreased from 61.42 (8.46) to 53.19 (9.26) kg at 6 months after surgery with BMI decline from 22.49 (2.77) before operation to 19.45 (3.08) at 6 months after surgery. Fasting plasma glucose levels showed a significant decrease (P = 0.035) in the T2DM group at a six-month point of 7.00 (2.21) mmol/L compared to preoperative levels of 7.67 (2.32) mmol/L. HDL levels increased significantly in the Non-T2DM group at six months postoperatively at 1.52 (0.05) with P < 0.001 compared to preoperative levels of 1.22(0.04) mmol/L. TG, LDL, and TC levels decreased significantly in both groups from the preoperative to the 6-month point.

**Conclusions:**

Esophagectomy induces weight loss in T2DM and Non-T2DM groups, improves long-term glucose metabolism in the T2DM group, and enhances lipid metabolism in both groups. Further research is needed to understand their mechanisms.

Esophageal cancer ranks seventh globally for new male cases and thirteenth for females. It also ranks sixth in male deaths and ninth in female deaths. China significantly impacts these statistics, accounting for over half of the world's incidence and deaths at 53.7 and 55.3%, respectively [1]. Though the occurrence of esophageal cancer is declining in China, the country still has a high incidence of esophageal cancer, with noticeable regional variations [2]. Early detection and diagnosis of esophageal cancer remains an essential method of preventing and treating the development of esophageal cancer. Esophagectomy is a vital surgical procedure for treating esophageal cancer and is an integral component of a comprehensive cancer treatment plan. There are two main methods of esophagectomy: open surgery and minimally invasive surgery. Compared to open surgery, minimally invasive surgery results in less blood loss, fewer respiratory complications, and shorter hospital stays [3]. This technique is now commonly practiced in clinical settings [4].

Type 2 diabetes mellitus is a metabolic disorder marked by high plasma glucose levels, irregular lipids, and protein metabolism [5], a prevalent, chronic health condition affecting individuals worldwide. Its incidence is escalating at an alarming rate. An estimated 552 million people worldwide will be affected by T2DM in the 2030s [5]. The prevalence of T2DM has attained epidemic proportions and is projected to affect over 400 million individuals worldwide [6]. Moreover, T2DM increases mortality in esophageal cancer patients who undergo esophagectomy, and co-morbidities related to Type 2 Diabetes Mellitus (T2DM) such as arterial hypertension, dyslipidemias, cardiovascular disorders, liver lipidosis, and other metabolic syndromes have been recently explored in greater detail [7]. Many procedures, for instance, bariatric surgery and gastric cancer surgery involving the GI tract, effectively improve metabolic disorders in type 2 diabetes mellitus [8, 9]. It is still unclear if esophageal cancer, a type of gastrointestinal surgery, has any metabolic effects on patients undergoing cancer treatment. The link between Type 2 Diabetes Mellitus and the risk of esophageal cancer has been the subject of numerous investigations for the past two decades, spanning various contexts such as hospital and population studies. However, most of these studies have focused on the relationship between type 2 diabetes and postoperative complications [10] rather than the metabolism changes.

Patients with diabetes need specific preoperative preparation and careful postoperative care. Regulating their blood glucose levels and other metabolic markers is crucial for optimal postoperative recovery and long-term health. The rising number of diabetes patients highlights the need to clarify the impact of diabetes on the prognosis following surgery for esophageal cancer.

## Materials and methods

### Patient selection and outcome

To investigate the impact of esophagectomy on the metabolic profile of individuals with diabetes, and to discern potential divergences in outcomes between diabetic and non-diabetic cohorts, this study involved 62 diabetes patients and 152 non-diabetes patients subjected to esophageal surgery in the Department of Cardiothoracic Surgery, the 900th Hospital of Joint Logistic Support Force from 2017 to 2022. All patients were followed for six months after their surgery. There were 46 male patients, with a mean age of 47.93 ± 9.24 years old, and 16 female patients, with a mean age of 44.19 ± 9.33 years old in the group. The mean preoperative body mass index (BMI) was 23.23 ± 2.94 kg/m2, and the mean body weight mass was 64.01 ± 10.49 kg—preoperative patients’ characteristics shown in Table 1.

**Table 1:** Characteristics of 214 patients with or without T2DM

Characteristics	Before matching			After matching		
	T2DM n = 62	Non-T2DM n = 152	P value	T2DM n = 52	Non-T2DM n = 52	P value
Age	63.81 (8.59)	59.87 (9.48)	0.005*	62.69 (8.05)	63.21 (8.86)	0.755
< 45	0	6 (3.95%)		0	1 (1.92%)	
45–55	10 (16.13%)	43 (28.29%)		9 (17.31%)	8 (15.38%)	
55–65	20 (32.36%)	53 (34.87%)		19 (36.54%)	20 (38.46%)	
65–75	24 (38.71%)	39 (25.66%)		20 (38.46%)	17 (32.69%)	
75–80	8 (12.90%)	7 (4.61%)		4 (7.69%)	3 (5.77%)	
≥ 80	0	4 (2.63%)		0	3 (5.77%)	
Sex			0.902			0.631
Male	46 (74.19%)	115 (75.66%)		40 (76.92%)	42 (80.77%)	
Female	16 (25.81%)	37 (24.34%)		12 (23.08%)	10 (19.23%)	
pN category			0.191			0.607
N0	34 (54.84%)	85 (55.92%)		29 (55.77%)	30 (57.69%)	
N1	17 (27.42%)	33 (21.71%)		13 (25.00%)	13 (25.00%)	
N2	10 (16.13%)	21 (13.82%)		10 (19.23%)	7 (13.46%)	
N3	1 (1.61%)	13 (8.55%)		0	2 (3.85%)	
pT category			0.067			0.066
T1	18 (29.03%)	22 (14.47%)		14 (26.92%)	8 (15.38%)	
T2	17 (27.42%)	45 (29.61%)		12 (23.08%)	23 (44.23%)	
T3	26 (41.94%)	81 (53.29%)		26 (50.00%)	21 (40.38%)	
T4	1 (1.61%)	4 (2.63%)		0	0	
Tumor staging			0.693			0.656
Stage I	12 (19.35%)	22 (14.47%)		11 (21.25%)	8 (15.38%)	
Stage II	29 (46.77%)	76 (50.00%)		24 (46.15%)	28 (53.85%)	
Stage III	21 (33.87%)	54 (35.53%)		17 (32.69%)	16 (30.77%)	
Type of surgical procedure			0.539			0.438
Open	8 (12.90%)	26 (17.11%)		7 (13.46%)	11 (21.15%)	
Minimally invasive	54 (87.10%)	126 (82.89%)		45 (86.54%)	41 (78.85%)	
BMI	23.16 (2.99)	21.39 (0.24)	< 0.001*	22.67 (2.90)	22.48 (2.77)	0.736
< 18	3 (4.84%)	15 (9.87%)		3 (5.77%)	1 (1.92%)	
18–24	36 (58.06%)	109 (71.71%)		34 (65.38%)	35 (67.31%)	
24–28	19 (30.65%)	25 (16.45%)		13 (25.00%)	14 (26.92%)	
> 28	4 (6.45%)	3 (1.97%)		2 (3.85%)	2 (3.85%)	
Smoking			0.399			0.825
Yes	15 (24.19%)	29 (19.08%)		13 (25.00%)	15 (28.85%)	
No	47 (75.81%)	123 (80.92%)		39 (75.00%)	37 (71.15%)	
Drinking			1			0.813
Yes	14 (22.58%)	44 (28.95%)		11 (21.15%)	12 (23.08%)	
No	48 (77.42%)	108 (71.05%)		41 (78.85%)	40 (76.92%)	
Regular exercise			0.449			0.617
0–3	14 (22.58%)	24 (15.79%)		12 (23.08%)	10 (19.23%)	
3–7	27 (43.55%)	77 (50.66%)		22 (42.31%)	27 (51.92%)	
7–10	21 (33.87%)	51 (33.55%)		18 (34.62%)	15 (28.85%)	
Diet score			0.250			0.925
0–3	17 (27.42%)	27 (17.76%)		14 (26.92%)	13 (25.00%)	
3–7	29 (46.77%)	75 (49.34%)		23 (44.23%)	25 (48.08%)	
7–10	16 (25.81%)	50 (32.89%)		15 (28.85%)	14 (26.92%)	

*T2DM* type 2 diabetes mellitus

Variables are expressed as the mean (SD), n (%)

*BMI* body mass index, *pN* pathological node, *pT* pathological tumor

*P < 0.05

All study subjects fulfilled the criteria to be eligible for esophageal surgery. Inclusive criteria: (1) Patients with esophageal cancer diagnosed by pathological examination through gastroscopic biopsy; (2) Patients with type 2 diabetes who were diagnosed with fasting plasma glucose levels greater than 7 mmol/L during hospital admission and/or a history of diabetes and/or the receiving treatment with diet, medication, or insulin [11]; (3) Complete tumor resection (R0 resection); (4) Detailed and complete case records are available. Exclusion criteria: (1) Previous or present malignant tumors; (2) Stage T4b and M1; (3) After new adjuvant treatment; (4) Unusual surgical method; (5) Significant dysphagia and major co-morbidity after surgery; (6) Neuropsychiatric illness; (7) Previous history of thoracic or abdominal surgery; (8) Coagulation disorders.

The Non-T2DM group categorized in this study had no preoperative co-morbidities of diabetes mellitus and other metabolic disorders; the diabetic patients in the T2DM group were those with a preoperative diagnosis of diabetes mellitus or were on medication and did not have other metabolic disorders. All diabetes diagnoses should be made based on the classification and diagnosis of diabetes released in 2016 [12]. All patients underwent a radical resection of esophageal cancer; Vital signs were stable, and patients and their families were informed and agreed.

### Surgery

Before the surgery, all patients underwent a multidisciplinary discussion and were found to meet the surgical criteria with no contraindications. The adoption of minimally invasive or open surgery is based on tumor characteristics, such as the tumor and surrounding tissue adjacency, lymph node size, and fusion, as well as the patient's personal history, for instance, whether there is a history of chest or abdominal surgery in the past. Minimally invasive surgery is performed under thoracic laparoscopy; open surgery is traditionally available. The operational mode has been presented in the earlier literature. Thoracic surgery involves the excision of the thoracic esophagus along with cleaning of para-esophageal lymph nodes, bilateral recurrent laryngeal nerve para-lymph nodes, submandibular lymph nodes, paratracheal lymph nodes, lower lung ligament lymph nodes, and paraphrenic lymph nodes as per AJCC guidelines. The abdominal part of the operation includes freeing the stomach, cleaning the abdominal lymph nodes according to the AJCC guidelines, and creating the stomach tube (with the linear cutting and closing device, we remove part of the slight curvature of the stomach, and the width of the stomach tube is about 3–6 cm). After separating the cervical esophagus through the incision in the neck, remove the esophagus and connect the upper esophagus and the stomach tube on the left side of the neck.

### Data collection

All data of patients were collected retrospectively, including general data (sex, age, previous medical history about diabetes, exercise and diet control), surgical data (tumor stage, type of surgical procedure, pN category, and pT category), and auxiliary examinations, which the case borrowing system can query. Fasting plasma glucose and weight were measured daily after admission, and they performed routine blood tests one week before the operation and one week, 1, 3, and 6 months (1 W, 1 M, 3 M, and 6 M) after the procedure. Lipid metabolism parameters include total cholesterol (TC), triglyceride (TG), high-density lipoprotein (HDL), and low-density lipoprotein (LDL), and these were recorded before the operation, 1 and 6 months (1 M, and 6 M) after the surgery.

The Diabetes Self-Management Questionnaire (DSMQ) [13] comprises 16 items, which collectively form a comprehensive psychometric assessment. The questionnaire includes four subscales, namely "Glucose Management", "Dietary Control", "Physical Activity", and "Health Care Use".

In this study, we have selected four items from the "Dietary Control" subscale and three items from the "Physical Activity" subscale, intending to evaluate patients' dietary and exercise behaviors. Scoring of the questionnaire involved reversing negatively worded items such that higher values are indicative of more effective self-care. The resulting scores were indicative of the participants' level of effectiveness in engaging in self-care practices. The evaluation scores of the patients have been transformed to a scale of 0–10 based on their actual score (raw score / theoretical maximum score *10; for example, for the subscale ‘Dietary Control’ a raw score of 6 leads to a transformed score of 6/12 × 10 = 5).

The remission of T2DM was divided into three situations: complete remission, partial remission, and no remission [8, 14]. Complete remission was defined as follows: fasting blood glucose (FBG) returned to a normal range with no requirement for medication. Partial remission was defined as follows: FBG returned to an improved level with reduced medication. No remission was defined as no changes in medicine, more medication requirements, or aggravation in FBG levels after surgery.

#### PSM

To minimize the selection bias of patients, PSM was conducted between the T2DM group and the non-T2DM group. Nearest neighbor matching was performed at a 1:1 ratio without allowing replacement, while a caliper width of 0.02 standard deviations was specified. The baseline information was matched, including age, sex, BMI, Type of surgical procedure, pN category, pT category, tumor staging, smoking, drinking, exercise, and diet control. Detailed preoperative patients' characteristics are shown in Table 1.

### Statistical analysis

Analysis of changes in body weight includes the difference in absolute weight loss and body mass index (BMI). Indicators of type 2 diabetes mellitus (T2DM) have the total change in fasting plasma glucose (FPG) and lipid levels and the analysis of nutritional indexes regarding fundamental differences.

The statistical analysis of Windows was performed using IBM SPSS software, version 26.0, supplied by IBM Corp, located in Armonk, NY. Continuous variables are presented as mean ± SD, and a t-test for independent samples determined the difference between the T2DM and Non-T2DM groups. The statistical significance was established with a p-value of less than 0.05. We analyzed diverse time points in the same set using a paired-sample t-test. For non-normal distribution, we used the Wilcoxon signed-rank test. GraphPad Prism version 9.5 is a software tool used for creating graphs. The key clinical features of the participants are displayed as percentages and total number of cases.

## Results

### Patient information before PSM

According to the inclusion and exclusion criteria, 214 patients were enrolled in the current study. Table 1 displays the fundamental traits of all the patients. There were 62 T2DM patients and 152 Non-T2DM patients. The patient's clinical characteristics, such as age, BMI, sex, pN category, pT category, tumor staging, type of surgical procedure, smoking, alcohol consumption, exercise, and diet control after esophagectomy, are summarized in Table 1, and these figures were compared between the two groups. T2DM group had higher age (p = 0.005 < 0.05) and BMI (p = 0.000 < 0.05). No significant difference was found in sex, pN category, pT category, tumor staging, type of surgical procedure, smoking, and drinking (p > 0.05) (Table 1).

### PSM analysis

There were differences between the T2DM group and the Non-T2DM group. Therefore, PSM was conducted to minimize the difference. After 1:1 PSM, 62 patients in the T2DM group and 152 patients in the Non-T2DM group were matched for final analysis. There was no difference in terms of age, sex, BMI, pN category, pT category, tumor staging, type of surgical procedure, smoking, drinking, exercise, and diet control after matching (p > 0.05) (Table 1).

After selection, 52 patients with T2DM were selected. In this study, 61.54% of patients had T2DM remission after esophagectomy. T2DM duration, anti-diabetes medication, and T2DM status are shown in Table 2.

**Table 2:** Clinical characteristics of patients with concurrent esophageal cancer and T2DM

Characteristics		No. 52
Age (years)		62.69 (8.05)
BMI (kg/m2)		22.67 (2.90)
Sex		
	Male	40 (76.92%)
	Female	12 (23.08%)
T2DM duration		
	< 5	15 (28.85%)
	5–10	14 (26.92%)
	> 10	23 (44.23%)
Anti-diabetes		
	Non	30 (57.69%)
	Oral hypoglycemic agent only	18 (34.62%)
	Insulin (with or without oral hypoglycemic agent)	14 (26.92%)
T2DM status 6 months after esophagectomy		
	No remission	20 (38.46%)
	Partial remission	18 (34.62%)
	Complete remission	14 (26.92%)

*T2DM* type 2 diabetes mellitus, *BMI* body mass index

Variables are expressed as the mean (SD), n (%)

### Weight and BMI

Over the 6-month follow-up, we recorded a considerable weight and BMI reduction in both T2DM and Non-T2DM groups. Postoperative weight and BMI changes were similar in the T2DM and Non-T2DM groups, demonstrating substantial and consistent reductions.

In the T2DM group, at one week, the body weight of the participants decreased to 62.10 (10.06) kg (p < 0.001), with a decrease of BMI to 22.32 (2.94). In the first, third, and 6th months after surgery, participants consistently experienced a reduction in body weight alongside a similar decrease in BMI. Specifically, one month after surgery, the weight decreased to 58.83 (8.74) kg (p < 0.001) with a BMI of 21.16(2.51). Furthermore, three months after surgery, the weight dropped to 58.26 (9.34) kg (p < 0.001), with a BMI of 20.89(2.86). After six months, the weight further decreased to 55.10(11.60) kg (p < 0.001), with a BMI of 19.77(3.48).

In the Non-T2DM group, weight decreased from 61.42 ± 8.46 preoperatively to 60.25 ± 8.34 (p < 0.001) after one week, to 56.49 ± 9.19 (p < 0.001) after one month, to 54.74 ± 7.90 (p < 0.001) after three months and to 53,19 after six months. As for BMI, on the seventh day following surgery, the mean BMI was 22.01 ± 2.75 kg/m2, indicating a significant decrease (p < 0.001). Substantial reductions in BMI were also observed in these patients during the first, third, and sixth months after surgery, respectively at 20.71 ± 2.80 (p < 0.001), 20.07 ± 2.71 kg/m2 (p < 0.001) and 19.45 ± 3.08 kg/m2 (p < 0.001).

**Fig. 1:**
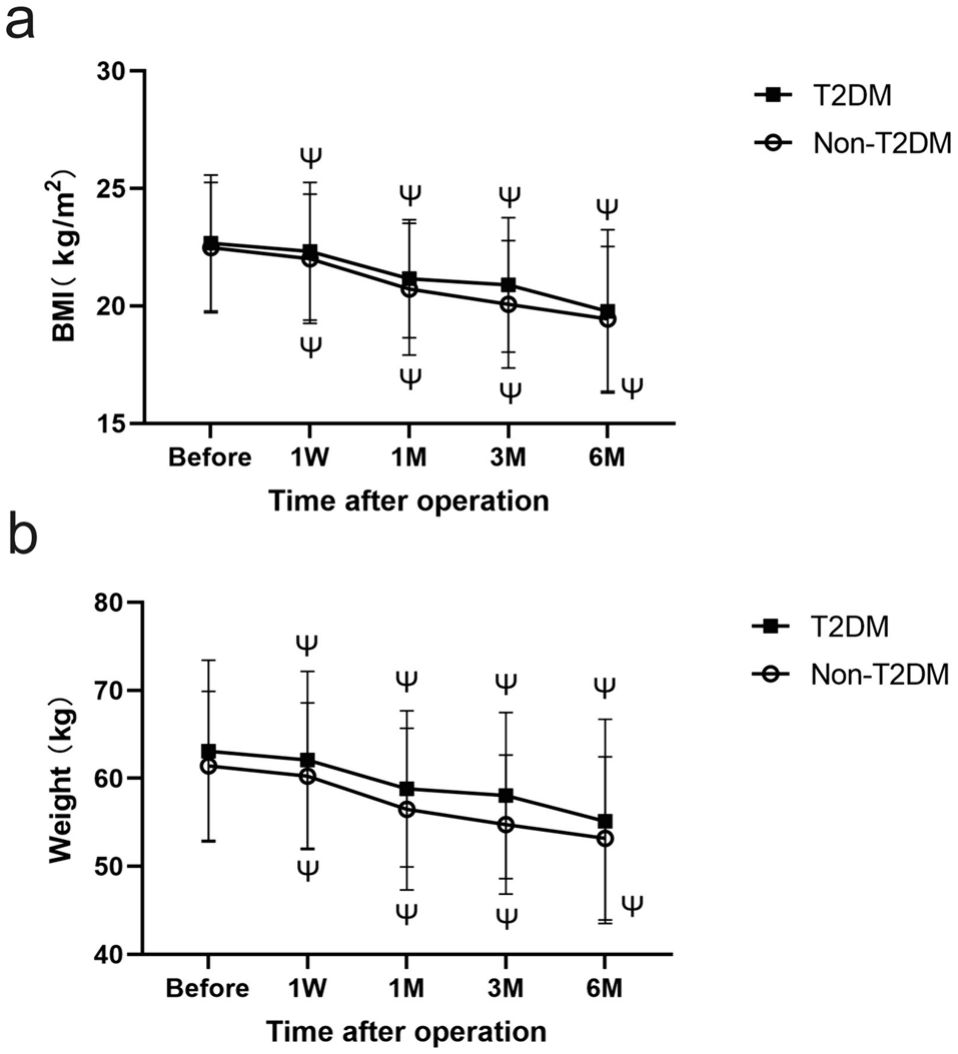
Changes in weight and body mass index (BMI) from preoperative point to 7 days, 1 month, 3 months, and 6 months postoperative of T2DM and Non-T2DM group and every postoperative moment both in Weight and BMI had witnessed a significant general decline compared to the preoperative point. Ψ means P < 0.001

Figure 1 and Table 3 present the time-based changes in Weight and BMI of two groups.

**Table 3:** Patient characteristics after surgery

	Time	T2DM(n = 52)						Non-T2DM(n = 52)			
BMI (kg/m2)	Before	22.67 (2.90)						22.49 (2.77)			
	1W	22.32 (2.94)				P < 0.001		22.01 (2.75)			P = 0008
	1 M	21.16 (2.51)				P < 0.001		20.71 (2.80)			P < 0.001
	3 M	20.89 (2.86)				P < 0.001		20.07 (2.71)			P < 0.001
	6 M	19.77 (3.48)				P < 0.001		19.45 (3.08)			P < 0.001
Weight (kg)	Before	63.10 (10.31)						61.42 (8.46)			
	1W	62.10 (10.06)				P < 0.001		60.25 (8.34)			P = 0.03
	1 M	58.83 (8.87)				P < 0.001		56.49 (9.19)			P < 0.001
	3 M	58.06(9.43)				P < 0.001		54.74 (7.90)			P < 0.001
	6 M	55.10 (11.60)				P < 0.001		53.19 (9.26)			P < 0.001
Fasting plasma glucose (mmol/L)	Before	7.67 (2.32)						5.18 (0.66)			
	1W	11.98 (4.96)			P < 0.001			7.63 (2.83)		P < 0.001	
	1 M	7.53 (2.85)			P = 0.741			6.07 (2.68)		P = 0.023	
	3 M	7.11 (2.61)			P = 0.188			5.57 (1.48)		P = 0.075	
	6 M	7.00 (2.21)			P = 0.035			5.31 (1.50)		P = 0.543	
Total cholesterol (mmol/L)	Before	4.80 (0.86)						4.58 (0.09)			
	1 M	3.80 (1.15)		P < 0.001				4.11 (0.13)		P = 0.001	
	6 M	4.48 (0.94)		P = 0.007				4.34 (0.11)		P = 0.025	
Triglycerides (mmol/L)	Before	1.36 (0.72)						1.07 (0.06)			
	1 M	1.27 (0.58)	P = 0.283				1.08 (0.05)		P = 0.859		
	6 M	1.07 (0.86)	P = 0.02				0.96(0.04)		P = 0.042		
HDL (mmol/L)	Before	1.21 (0.37)						1.22(0.04)			
	1 M	1.01 (0.31)	P < 0.001				1.22 (0.06)		P = 1.00		
	6 M	1.28 (0.40)	P = 0.2				1.52 (0.05)		P < 0.001		
LDL (mmol/L)	Before	3.17 (0.74)						3.11(0.09)			
	1 M	2.55 (0.83)	P < 0.001				2.74 (0.09)		P = 0.001		
	6 M	2.76 (0.74)	P = 0.001				2.63 (0.10)		P < 0.001		

*T2DM* type 2 diabetes mellitus, *BMI* body mass index, *HDL* high density lipoprotein, *LDL* low density lipoprotein

Data are presented as mean(SD)

### Fasting plasma glucose

The research findings revealed the long-term effects of reduced blood glucose levels in the T2DM group. The trend of glycemic changes was similar in the T2DM and Non-T2DM groups. However, after six months of surgery, the T2DM group witnessed a significant decrease compared to the level before the surgery, and the Non-T2DM group showed no significant difference from the station before the surgery.

The average preoperative blood glucose level in the T2DM group was 7.67 (2.32) mmol/L, which increased to 11.98 (4.96) mmol/L on the seventh day after surgery (p < 0.001). Further reduction in FPG levels persisted throughout the remaining observation period. The average FPG level declined to 7.53 (2.85) mmol/L after one month of surgery (P = 0.741), to 7.11 (2.61) mmol/L after three months (P = 0.188), and further to 7.00 ± 2.21 mmol/L during the sixth month after surgery (P = 0.035).

There was no significant reduction of fasting plasma glucose in the long-term observation in the Non-T2DM group. The first week witnessed an escalation from 5.18(0.66) to 7.62(2.83) mmol/L, then declined gradually to 6.07 (2.68) in one month (P = 0.023), to 5.57 (1.48) in three months (P = 0.075) and 5.31 (1.50) in 6th month (P = 0.543).

**Fig. 2:**
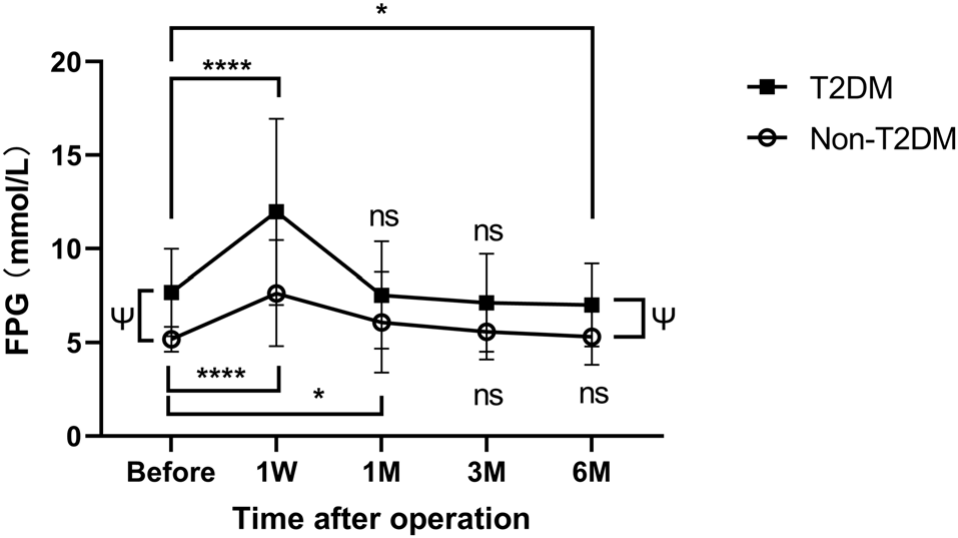
Changes of fasting plasma glucose (FPG) in the T2DM and Non-T2DM groups in preoperative point and 1 week, 1 month, 3 months, and 6 months after receiving esophagectomy with comparison of FPG between two groups. The T2DM group displayed an ongoing reduction in FPG 6 months after surgery while there was no comparable change in the Non-T2DM group. Ψ means P < 0.001, ****means P < 0.005, *means P < 0.05, and “ns” means P > 0.05

Figure 2 and Table 3 show the changes of FPG in two groups.

### Lipid profile

**Fig. 3:**
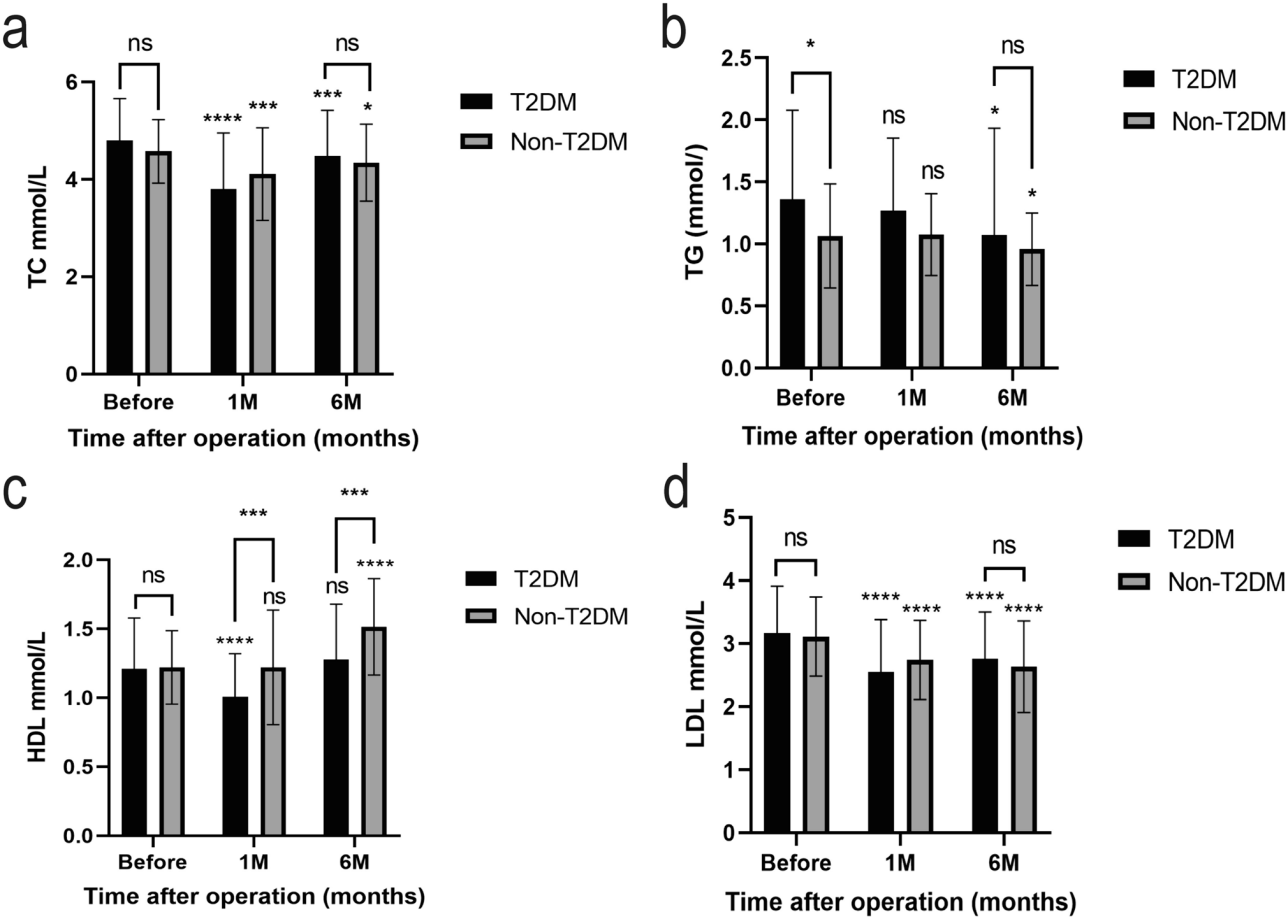
Lipid changes and comparison between T2DM and Non-T2DM groups before and 1 month, 6 months post-esophagectomy. Graphs from “a” to “d” show TC(total cholesterol), TG(triglyceride), HDL(high-density lipoprotein), and LDL(low-density lipoprotein) changes and group comparisons, respectively. “ns” means P > 0.05, ****means P < 0.005, ***means P < 0.01, *means P < 0.05

There was no significant difference in total cholesterol (TC) levels between the two groups before the surgery. After six months, both groups experienced a significant decrease in TC, with no observed difference (Fig. 3a). The TC level in the T2DM group before surgery (4.80 ± 0.86) mmol/L decreased to 3.80 ± 1.15 mmol/L in the first month (P < 0.001). During the sixth month after surgery, the level reached 4.48 ± 0.94 mmol/L (P = 0.007). While in the Non-T2DM group, the TC level decreased from 4.58 ± 0.09 to 4.11 ± 0.13 mmol/L (P = 0.001) in one month and then reached 4.34 ± 0.11 mmol/L (P = 0.025) after six months (Table 3).

As for triglycerides (TG), though preoperative levels of TG were significantly higher in the T2DM group than in the Non-T2DM group, six months after the surgery, the levels of TG decreased in both groups, and the difference between them disappeared (Fig. 3b). A statistically significant reduction of TG levels in the T2DM group was observed in the sixth month (1.07 ± 0.86 mmol/L, P = 0.02) when compared to the preoperative values (1.36 ± 0.72 mmol/L), while the first month did not reach a significance (1.27 ± 0.58, P = 0.283). Also, the Non-T2DM group witnessed a significant decline in the sixth month (0.96(0.04) P = 0.042), whereas the first month did not show a significance (1.08 ± 0.05, P = 0.859). Detailed information is shown in Table 3.

Additionally, there was no significant difference in HDL levels between the two groups at the preoperative level. After esophagectomy, HDL levels in the Non-T2DM group significantly increased six months postoperatively compared to the preoperative level. However, the T2DM group showed no significant change in HDL levels compared to preoperative. However, at six months postoperatively, the levels of HDL were notably more outstanding in the Non-T2DM cohort compared to the T2DM group (Fig. 3c). There was a significant increase of HDL levels in the Non-T2DM group in the sixth month with 1.52 (0.05), P < 0.001, compared to the preoperative measurement of 1.22(0.04) mmol/L. However, the first month was insignificant (1.22 ± 0.06, P = 1.00). Concerning the T2DM group, on the seventh day, the initial measurement indicated a decrease in HDL levels from 1.21(0.37) to 1.01 (0.31) mmol/L (P < 0.001). Subsequent measurements in the sixth month exhibited no significance (1.28 ± 0.40, P = 0.2) compared with the preoperative levels.

The last measured lipid fraction was low-density lipoprotein (LDL). LDL was not significantly different between the two groups preoperatively, and LDL showed a significant decrease in both groups six months postoperatively, but there was no significant difference between the two. All changes in LDL showed statistical significance (Fig. 3d). In the T2DM group, LDL levels decreased from the initial value of 3.17(0.74) mmol/L to 2.55 (0.83) mmol/L (P < 0.001) on the seventh day and reached 2.76(0.74) mmol/L (P = 0.001) in the sixth month. As for the Non-T2DM group, a gradual decline was observed from the preoperative level of 3.11 (0.09) mmol/L to 2.74 (0.09) mmol/L P = 0.001 in the first week and to 2.63 (0.10) mmol/L P < 0.001 in the 6th month (Table 3).

## Discussion

This retrospective study has shown a significant reduction in body weight and BMI and prolonged benefit on blood glucose levels and plasma lipid levels for patients with esophageal cancer and type 2 diabetes after receiving esophagectomy. Our study may guide postoperative metabolic management and instruct the medical use in patients with T2DM after esophagectomy.

Esophagectomy can positively reduce the weight and BMI of people with or without type 2 diabetes. In two groups, most patients lose over 12% of their weight and BMI within six months of esophagectomy. This result suggests that patients may be at high nutritional risk according to ESPEN (European Society for Clinical Nutrition and Metabolism) guidelines [15]. As a result, malnutrition and weight loss after esophagectomy is a significant problem [16].

According to previous research conducted by Anna Schandl [17], about 36% of patients experienced a body weight loss of ≥ 15% six months after undergoing esophageal surgery; Yuto Kubo [18] conducted a study that revealed that patients with esophageal cancer lost an average of 9.3% of their body weight three months after surgery, and 10.8% at six months after the procedure; D'Journo [19] reported that 55% of the survivors experienced a weight loss rate of more than 10% after the esophagectomy in their study of 205 patients who were 1-year disease-free survivors after surgery. Although most previous studies have shown that esophagectomy leads to weight loss, the focus has been primarily on patients without chronic metabolic co-morbidities. Our study indicates significant weight and BMI loss among patients in both T2DM and Non-T2DM groups after esophageal surgery, with a decrease of 1.34% in one week, 5.15% in one month, 9.71% in three months, and 12.39% in the sixth month and with longer follow-up weight and BMI decreased continuously. There are several reasons patients get a reasonable reduction of weight and BMI after esophageal cancer surgery: The present results indicate that gastro-esophageal symptoms such as appetite loss and eating difficulties significantly contribute to weight loss. Marti's research revealed a possible link between weight loss and appetite loss [16]. Martin L's study suggested that patients who experienced weight loss after esophagectomy reported clinically significant and statistically worse symptoms, such as eating difficulties, pain, fatigue, nausea, vomiting, and appetite loss [20]. Weight loss may also be related to changes in gastrointestinal hormones. Similar research by Elliott suggested that decreasing GLP-1 may reduce hunger early after surgery, leading to weight loss [21].

Plasma lipid levels in patients with or without T2DM show a positive decrease after esophagectomy. In this study, we retrospectively analyzed the lipid levels of patients with esophageal cancer with T2DM or Non-T2DM who had undergone esophagectomy six months earlier. We found that six months after surgery, the patient's total cholesterol (TC), low-density lipoprotein (LDL), and triglyceride (TG) levels showed a significant decrease in long-term follow-up in both two groups, while HDL levels increased significantly in the sixth month only in the Non-T2DM group. A similar tendency of TC, TG, and LDL was also observed in the T2DM group, while HDL of the T2DM group showed no significant change six months after surgery.

There was no significant difference in preoperative TG levels between the two groups, and at six months, the two groups showed a significant decrease in TG levels compared to their respective preoperative levels, whereas there was still no significant difference between the two groups. This suggests that esophagectomy reduces postoperative TG levels in patients with and without diabetes mellitus, but no significant difference in the effect on the two groups was observed.

After esophagectomy, HDL levels in the Non-T2DM group significantly increased 6 months postoperatively compared to the preoperative level. However, the T2DM group showed no significant change in HDL levels compared to preoperative. Additionally, there was no significant difference in HDL levels between the two groups at the preoperative level. However, at six months postoperatively, HDL levels were significantly higher in the Non-T2DM group than in the T2DM group. LDL was not entirely different between the two groups preoperatively, and LDL showed a significant decrease in both groups six months postoperatively, but there was no significant difference between the two. The TG and LDL levels trend was similar to TC; Table 3 and Fig. 3 show the detailed information. The changes in three lipid metabolism indices showed a positive control of lipid metabolism. By the sixth month, HDL levels are significantly higher than before surgery—the increased HDL level at the long-term treatment point signals improved lipid metabolism.

Researchers Elliott et al. have conducted a study [22] that demonstrates that patients who underwent esophagectomy experience an exaggerated gut hormone response. This response was observed through changes in GLP-1 responsiveness, which has been linked to postoperative satiety and weight loss. Additionally, Nicholas RS Stratford et al. [23] have suggested that changes in gut hormone signaling after surgery for esophagogastric cancer play a vital role in weight loss among patients after esophagogastric cancer surgery. Gut hormone signaling can affect food intake through various pathways and, as a result, may be associated with malnutrition in patients after esophagogastric cancer surgery. This association may explain the reduced lipid metabolism observed in the patients in this study.

The following reasons can be suggested for the alterations in plasma lipid levels: Patients who have undergone esophagectomy may experience a reduction in their lipid levels due to postoperative gastro-esophageal symptoms, including appetite loss, eating difficulties, and changes in gut hormones, which result in decreased food intake and consequently reduced body weight [16, 21].

Esophagectomy has long-term effects on fasting plasma glucose levels in patients with esophageal cancer and type 2 diabetes. Diabetes patients have an abnormal blood sugar metabolism. Our research has shown that the mean fasting plasma glucose level significantly differs from preoperative levels in the long term. The research findings revealed the long-term effects of reduced blood glucose levels in the T2DM group. The trend of glycemic changes was similar in the T2DM and Non-T2DM groups. However, after six months of surgery, the T2DM group witnessed a significant decrease compared to the level before the surgery, and the Non-T2DM group showed no significant difference from the level before the surgery. Fasting plasma glucose showed a substantial decrease after six months (7.07 ± 2.12, P = 0.045) compared to the preoperative level (7.63 ± 2.22), while no significant change was observed in post-prandial insulin response during the early postoperative period. This conclusion coincides with the findings of numerous previous investigations.

Long-term reduction in blood glucose levels may be associated with changes in insulin and gastrointestinal hormones. The study conducted by Elioot et al. [22] has suggested that changes in gut hormones, particularly GLP-1, along with insulin response, correlate with postoperative glycemic changes in patients who underwent esophagectomy. Similarly, Miyazaki et al. [24] have found that the plasma ghrelin decreased significantly on postoperative day 7, and gradually recovered thereafter. They have also noted that the plasma ghrelin has been linked to postoperative body weight loss, which contrasts with the glycemic changes reported in the current study. Our research, however, has observed that the trend of plasma ghrelin was opposite to that of fasting plasma glucose, so we hypothesized that plasma ghrelin was associated with changes in post-operative fasting plasma glucose levels.

Postoperative hyperglycemia is found on the seventh day and may be associated with stress and infection. Diabetic patients with surgical co-morbidity may experience stressful hyperglycemia due to increased glucagon (glucocorticoids) levels in the body. [25, 26]; Additionally, transient postoperative hyperglycemia may induce an oxidative stress response leading to elevated blood glucose [27, 28].

Based on previous studies, we hypothesize that there may be two primary mechanisms by which esophagectomy improves glucose levels in type 2 diabetes:(1) gastrointestinal rerouting caused by esophagectomy also significantly affected gastric emptying and altered gastrointestinal hormone (especially GLP-1) secretion, and (2) Changes of plasma ghrelin following esophagectomy lead to reduced eating behavior and weight loss, which affects glucose metabolism. Further studies are needed to explain the mechanism of these results.

This study has three shortcomings. To begin with, this is a retrospective study conducted in a single center with a small eligible sample size of only 104 patients with esophageal cancer combined with or without type 2 diabetes, which accounts for approximately 10% of the patients who underwent esophageal cancer surgery during the same period. Additionally, some patient data were lost due to trend matching, which could lead to bias. Hence, a more extensive multicenter study is required to verify our conclusions. The Propensity Score Matching (PSM) technique has helped reduce the differences between groups of diabetic patients. However, it is important to note that variations still exist despite the application of this technique. Therefore, it is recommended to use larger samples that encompass a wider range of influencing factors to refine the findings even further. This approach could significantly enhance the reliability and accuracy of the information gathered, making it more beneficial for future research. Thirdly, the study does not include indicators related to glycosylated hemoglobin (HbA1c) and blood glucose, which could impact the decision. Not all patients with diabetes in our unit are tested for HbA1c; tests are only conducted when patients complain of poor blood glucose control or have high initial blood glucose levels upon admission. Thoracic surgeons pay more attention to immediate blood glucose control during the perioperative period than HbA1c, which reflects the overall situation of blood glucose control over a short period.

Hyperlipidemia and diabetes mellitus have been confirmed to be relevant and independent risk factors for the postoperative recurrence of esophageal cancer. The higher fasting plasma glucose levels, insulin resistance, and total cholesterol are related to the earlier postoperative recurrence time [29]. Postoperative hyperglycemia is an independent predictor of the development of surgical infections, anastomotic fistulas, and prolonged hospital stays, and the higher the blood glucose level, the higher the risk of its development [30, 31]. The metabolic changes in patients with esophageal cancer and diabetes mellitus after surgery have not been studied. Therefore, we aimed to investigate the postoperative changes in weight and glucolipid metabolism in this group of patients. Our study confirms the beneficial effect of esophagectomy on weight and lipid levels in patients with type 2 diabetes. Optimal postoperative metabolic management is of paramount importance for diabetic patients undergoing esophagectomy, as it has a significant impact on their recovery and overall quality of life. Therefore, it is strongly recommended that patients and healthcare professionals alike prioritize metabolic management during the postoperative period. Postoperative metabolic management has a significant impact on the recovery and quality of life of diabetic patients undergoing esophagectomy. Therefore, we urge both patients and clinicians to pay close attention to metabolic management during the postoperative period.
